# Intrinsic and environmental factors modulating autonomous robotic search under high uncertainty

**DOI:** 10.1038/s41598-021-03826-3

**Published:** 2021-12-31

**Authors:** Carlos Garcia-Saura, Eduardo Serrano, Francisco B. Rodriguez, Pablo Varona

**Affiliations:** Grupo de Neurocomputación Biológica, Dpto. de Ingeniería Informática, Escuela Politécnica Superior, Universidad Autónoma de Madrid, 28049 Madrid, Spain

**Keywords:** Engineering, Planetary science, Ocean sciences, Power law, Scale invariance

## Abstract

Autonomous robotic search problems deal with different levels of uncertainty. When uncertainty is low, deterministic strategies employing available knowledge result in most effective searches. However, there are domains where uncertainty is always high since information about robot location, environment boundaries or precise reference points is unattainable, e.g., in cave, deep ocean, planetary exploration, or upon sensor or communications impairment. Furthermore, latency regarding when search targets move, appear or disappear add to uncertainty sources. Here we study intrinsic and environmental factors that affect low-informed robotic search based on diffusive Brownian, naive ballistic, and superdiffusive strategies (Lévy walks), and in particular, the effectiveness of their random exploration. Representative strategies were evaluated considering both intrinsic (motion drift, energy or memory limitations) and extrinsic factors (obstacles and search boundaries). Our results point towards minimum-knowledge based modulation approaches that can adjust distinct spatial and temporal aspects of random exploration to lead to effective autonomous search under uncertainty.

## Introduction

Animals routinely perform highly efficient search tasks associated with foraging, mating, the location of breeding or shelter sites, etc. The success of these tasks is sustained even when they are carried out under high uncertainty conditions where random search is involved^1–3^. Animals use different senses that provide information to continuously modulate their search behaviour, particularly when the level of uncertainty is large. Each sense, e.g., olfactory, visual, auditory, tactile, has a different range and animals smoothly shift between them and combine information from different sources to drive their search strategies in high uncertainty situations.

**Figure 1:**
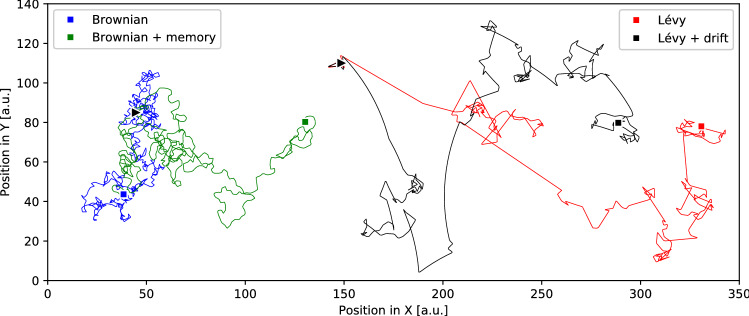
Brownian and Lévy search simulation models. Traces on the left are examples of planar Brownian exploration: The blue trace is a pure Brownian search and the green trace incorporates a small local memory of previous positions to avoid recently explored areas (see details in further sections). “Brownian + memory” used the same initialisation seed as the pure Brownian search, and both ran for $10^3$ steps. The overlapped representation allows to identify that incorporating a small memory leads to a more efficient use of the available resources, expanding the exploration clusters of the pure Brownian strategy. Traces on the right show a similar experiment applied to Lévy exploration with $\alpha =2.5$. The red trace is a pure Lévy search, and the black trace incorporates the effect of locomotion drift (0.6$^\circ$, implementation described below). The Lévy strategy with drift corresponds to situations where global position references are not available, since a robot cannot perform indefinite perfectly-straight trajectories without correcting from external information. Note the differences between the two Lévy searches: both start very close to each other (indicated by triangles) and gradually diverge when drift is present, with noticeable deviations during long walks (final positions indicated by squares). We study a set of intrinsic and extrinsic factors and show that their effect can be utilised to modulate low-informed autonomous random search.

The study of animal search behaviour has quantitatively characterised animal movements under sensory limitations which correspond to Brownian or correlated random walks and Lévy walk exploration (e.g., see for review^1,3–5^, see also Fig. 1). Different strategies provide distinct outcomes and success levels in the context of specific animal behaviours and habitat contexts. Beyond the controversy regarding the best characterisation of animal motion during random search behaviour^6–12^, random-like locomotion does exist in nature to overcome the problems of searching under uncertainty.

Random motion has been assessed theoretically and experimentally from different perspectives in the context of ecological, biological, physical and robotic studies (see for review^1,13–15^). Diffusive properties of random walks, where mean squared displacement linearly increases with time, and superdiffusive properties of Lévy walks, where mean squared displacement increases superlinearly^16,17^ are known mechanisms that nature uses to deal with low information levels from sensory systems.

Likewise, autonomous robotic search problems always face some level of uncertainty regarding the environment, sensor performance and reliability, the motor plant function, the possible location of the search targets, the latency in which they may appear/disappear, or move, etc. In those problems where uncertainty is large, and partial or no information is available to implement algorithmic or heuristic searches, strategies inspired in biology have been proposed to address them, both in single robots^18–21^ and in swarm strategies^22–27^.

It is important to note that animal search strategies under uncertainty are neither pure Brownian nor strictly compliant with a Lévy distribution, since real-world search behaviour is always restricted by body, energy or environmental constraints, and also by the continuous modulation of sensory input. Random walk strategies can also naturally emerge through the interaction from collective behaviours (e.g.^28,29^) or the environment (e.g.^30,31^). Autonomous robotic search strategies can benefit from an in-depth analysis of these aspects when implementing such bioinspired solutions in low-informed reconnaissance, surveillance, and exploration tasks with limited input data.

This paper studies the factors that modulate Brownian, naive ballistic and Lévy based robotic search in terms of their diffusive properties and, in particular, the spatial and temporal properties of the exploration. Among intrinsic factors are the motor plant (i.e., motion accuracy and drift), the inherent resources such as available energy (i.e., battery capacity or fuel level), sensor information (i.e., range, accuracy, intermodality fusion), the parameterisation of the search strategy itself, etc. Among extrinsic factors are the characteristics of the environment boundaries, obstacles, terrain features, atmospheric conditions, environment media type, mapping availability, etc. With the end goal of designing effective robotic strategies for high uncertainty situations, we study these intrinsic and extrinsic factors and how they can be taken into account with basic a priori information.

## Models and methods

In this section we explain the simulation models, the metrics and the representation methods used for describing the results of our study. The robot simulation platform is available as supplementary material for easy reproduction of the results and, more importantly, for their extension when exploring other intrinsic and extrinsic factors that modulate random search strategies under uncertainty.

### Search environments

Our study addresses the influence of intrinsic and extrinsic factors on random search strategies for autonomous robots. In order to compare the effectiveness of each strategy in different domains, a series of environment maps were defined representing the extrinsic factors affecting search under high uncertainty. Each map progressively incorporated distinct characteristics and varied features often found in real world domains:

- Plain: The global boundary was defined as a plain circumference, which represents distance-bound exploration of an area without obstacles.
- Triangle: A geometric map with acute angle corners, which represents a triangulated exploration patch.
- Forest: The Plain map with the addition of randomly distributed obstacles. Two densities were used: “Sparse Forest” and “Dense Forest”.
- Craters: The Plain map with the addition of two inaccessible regions, effectively creating two search areas interconnected by three passages.
- Corridor: A geometric map with very high aspect ratio, three distinct areas with narrow passes in between.

Table 1 summarises the implemented maps and their parameters. These representative environments allow analysing the effect of various physical interactions or virtual restrictions over low-informed search strategies. Note that differences in exploration area sizes could also play a fundamental role and affect the outcome. Our study tackles this problem by analysing the set of strategies for each independent map. The area parameter is further discussed in the conclusions, as it could also be studied with the tools provided.

**Table 1:** Representative search environments used in our quantitative study and their parameters.

Map name	Plain	Triangle	Sparse forest	Dense forest	Craters	Corridor
Geometry						
Parameters	*d*=100.0	*s*_*a*_=80.0 *s*_*b,c*_=89.4	*d*=100.0 *d*_*n*_=2.9 *N*=50	*d*=100.0 *d*_*n*_=2.9 *N*=100	*d*=100.0 *d*_1_=28.6 *d*_2_=38.5	*s*_*w*_=100.0 *s*_*h*_=14.3 *d*_1,2_=7.1
Area	7853	3180	7539	7246	6051	1159

Each map geometry is shown in black and white (explorable area and obstacles, respectively). Lengths and areas are given in a.u. (see “Robot simulation models” section). Parameter *D* indicates map diameter, $d_n$ indicates obstacle diameter, *N* indicates obstacle count, and $s_i$ indicates segment length.

### Robot simulation models

For our quantitative analysis we considered single-robot random walk models that range from pure Brownian motion to distinct uninformed and low-informed Lévy walks. The simulations were implemented in the *XY* plane of arbitrary length units (a.u.), specifying a robot collision diameter $d_c= 1$ a.u. with a sensory range diameter $d_s= 4$ a.u.. These parameters represent high uncertainty situations in which visibility is low and/or sensor modalities are range restrictive (olfactory/tactile sensors, buried targets, etc) in the context of the representative search environments described above. Simulation choices were implemented with the knowledge acquired from previous experiments using real world agents with artificial noses^24^. At each simulation step the robot advanced $s= 0.1$ a.u., and the random search strategy modulated the new direction of the robot $\theta (t)$. Straight-line *travels* of length *l* maintain the same robot direction, $\theta _{strategy}(t)=\theta (t-1)$ for the corresponding number of simulation steps $\lceil \frac{l}{s}\rceil$. Thus, coordinates of the robot at position $\mathbf {p}=(x,y)$ are updated at each time step *t* as:

1 $$\begin{aligned} \begin{array}{lccl} x(t)=x(t-1)+s \cdot \cos (\theta (t-1)) \\ y(t)=y(t-1)+s \cdot \sin (\theta (t-1)) \\ \theta (t)= \theta _{strategy}(t) + \theta _{drift} \end{array} \end{aligned}$$

where $\theta _{strategy}(t)$ is the modulated robot heading and $\theta _{drift}$ is the drift constant (discussed further in the text). A high-density square grid was employed to compute and analyse the exploration statistics, with a cell size of 0.1 a.u. The same grid is used to quantify the metrics of each exploration as grid cells store the revisit information and search statistics in a discretized manner, the shape of each map (boundaries and obstacles) defines what areas of the grid can be explored by the robots. Before committing to a particular step, each strategy considered the presence of boundaries or obstacles within the collision range, $d_c<2|\mathbf {p}-\mathbf {o_i}|$, where $\mathbf {o_i}$ is the position of the closest boundary or obstacle *i*. This situation was handled with the *bounce* strategies discussed below.

To implement the Lévy walk strategies, the robot moved to each new position with travels of length *l* drawn from a heavy-tailed distribution. In particular, the target length of each travel *l* was drawn from the following probability density function:

2 $$\begin{aligned} f(l) = \left\{ \begin{array}{lccl} {{\frac{\alpha - 1}{l_{min}}} \left( \frac{l}{l_{min}} \right) ^{-\alpha }} &{} \text{ for } &{} l \ge l_{min} \\ {0} &{} \text{ for } &{} l < l_{min} \end{array} \right. \end{aligned}$$

where $\alpha$ is the exponent parameter ($2<\alpha <3$) and $l_{min}$ is the lower bound parameter^13,32^. In the experiments reported below, we chose values for $\alpha$ that sustain the superdiffussion properties characteristic of Lévy search. The bound parameter was set to $l_{min}= 2.1$ a.u. so that in the shortest travels the robot moves just beyond its sensor range $d_s$. Between each complete travel, an in-place rotation was generated with a uniformly distributed angle $\theta _{strategy}(t)\in [0,360^\circ )$.

The simulations in this study also implemented Brownian search and ballistic (straight line) search. Brownian exploration was implemented as a random walker with travels of fixed length $l=l_{min}$ and uniform rotations $\theta _{strategy}(t)\in [0,360^\circ )$ as in the Lévy search. Ballistic exploration was implemented as a continuous forward motion (i.e., an infinite travel), which was only truncated when colliding with boundaries and obstacles. Figure 1 shows examples of Brownian and Lévy searches as generated with the above mentioned methods.

Low-informed search in bounded environments requires specific *bounce* strategies to deal with obstacles and boundaries. As illustrated throughout our study, small variations in these obstacle-handling routines can yield very different searches. We chose a representative set of bounce strategies that define the interaction of the robot with the environment. Upon a collision, the incidence angle is defined as $\theta _{incidence}(t)=\theta (t)-\theta _{o_i}(t)$, where $\theta (t)$ is the robot heading and $\theta _{o_i}(t)$ is the normal at the collision point at the boundary or obstacle $\mathbf {o}_i$:

- In “random bounce” the new robot direction is drawn from a uniform distribution *U* covering 180$^\circ$ away from the obstacle or boundary: $\theta _{strategy}(t)=\theta _{o_i}(t)+u_i$, where $u_i$ is a sample from $U(-90^\circ ,90^\circ )$.
- In “mirror bounce” the robot follows a specular/regular reflection, a “billiard ball” collision, $\theta _{strategy}(t)=\theta _{o_i}(t)+\theta _{incidence}(t)$. Note that mirror bounce and random bounce could be combined in a vast continuum of possibilities; in this study we show their effect separately.
- In “wall follow” upon collision the robot continues perpendicular to the obstacle or boundary, $\theta _{strategy}(t)=\theta _{o_i}(t) + 90^\circ \cdot \text {sign}(\theta _{incidence}(t))$, where the new direction follows the robot incidence angle.
- In “recast bounce”, specific of Lévy implementations, a new travel length *l* is computed at each collision. The travel is redrawn from model (2), and next, the “random bounce” strategy is applied to orient the robot away from the obstacle.

As another source of uncertainty, several simulations considered the presence of motion drift when implementing the trajectories, see $\theta _{drift}$ in model (1). While often disregarded, this is another factor that affects Lévy flight implementations^21,33^. Linear translations were approximated as arcs, and the drift parameter was specified as the total amount of rotation that a robot undergoes after moving for a given distance –the longitude of the performed arc–. Drift was quantified in degrees per arbitrary length unit (*deg*/*a*.*u*., abbreviated as $^\circ$), see also^21^. Note that this implementation tackles both the inherent systematic bias of the motor plant and, more importantly, the long term drift that arises when external position references become unavailable.

In “memory” labelled strategies, a model of short-term memory was implemented with the internal storage of the robot positions for the most recent $N=100$ simulation steps. The search strategies used this memory to reject trajectories that would lead to recently explored grid cells. We denote $\mathbf {q}$ the robot target position given by $(x,y,\theta _{strategy})$ and travel length *l*. Trajectories to the target position $\mathbf {q}$ were rejected when $\mathbf {q} \in \{\mathbf {p}_{t},\mathbf {p}_{t-1},\ldots ,\mathbf {p}_{t-N}\}$, and $\theta _{strategy}(t)$ was recast until an unexplored target was found. Note that the model was kept lean by computing target grid cells only, allowing for some overlap during the trajectories while still leading to unexplored areas. Also, to avoid “nowhere-to-go” situations, due to trapping by boundaries or obstacles, we set a probability of 5% chance of pursuing already visited locations. The simplicity of this memory allows for a straightforward implementation that can be robust in the absence of external position references. Basic dead reckoning navigation allows implementing the proposed short-term model, whereas larger memories could be realised by means of pheromone inspired labelling, wheel mark tracking, and overall more complex approaches.

For specific Lévy search strategies, there was an additional energy consideration referred to as “battery truncation”, where Lévy-distribution travels had a maximum length. This was set to 10% of the total available energy, approximated as the total distance that a real-world robot could move with a fully charged battery, 4493 a.u. estimated from a scaled robot model^24^.

Simulations for all strategies were initialized in the middle area of every map, using a random angle $\theta$. Further details on the implementation can be found in the provided software platform (Supplementary information).

### Search efficiency metrics

**Figure 2:**
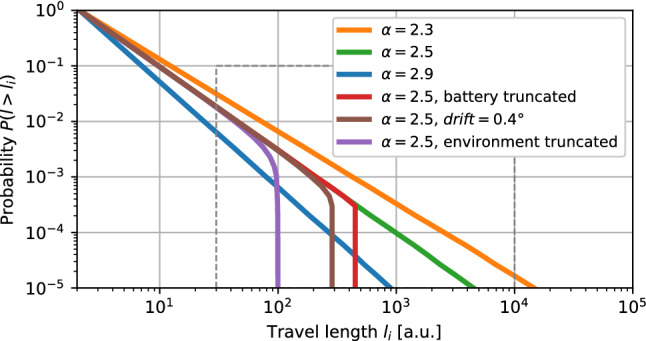
Modulation of Lévy travel length probability by intrinsic and extrinsic factors. Each represented trace is the complementary cumulative distribution function (CCDF) of the travel lengths for each strategy/parameter combination. We show its modulation by the exponent parameter $\alpha$ (blue, orange and green traces) and under different factors: intrinsic (battery truncated –red– and drift bound –brown–) and extrinsic (environment bound, a basic model that truncates trajectories at $10^2$ a.u. to represent search area diameter limit –purple trace–). Each CCDF was computed numerically with the models described in “Robot simulation models” section. The dashed rectangle indicates the region employed for the drift and battery analyses discussed in Fig. 3.

**Figure 3:**
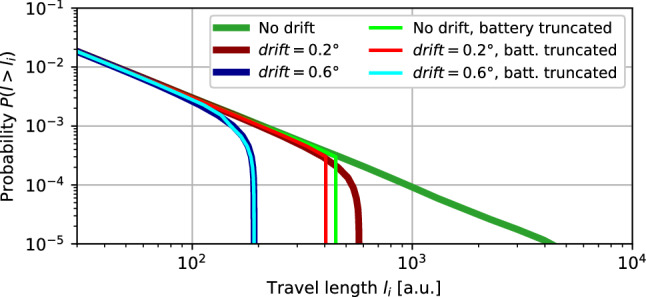
Effect of motion drift and battery constraints over travel length probability. For all traces in this figure $\alpha =2.5$ and no environment bounds. A pure implementation of Lévy search would strictly follow a power-law (dark-green trace). In practice, there are several intrinsic and extrinsic parameters that affect the planned strategy when implemented in a robot. Motion drift is caused by uncertainty in the locomotive plant (i.e., wheel slipping, inertial sensor accuracy, etc.) and effectively limits long travels. Two representative drift values are depicted in red and blue. Energy limitations are considered in the “battery truncated” case (brighter red, green and blue traces) where individual travels are limited in length to use at most 10% of the available battery (“Robot simulation models” section).

The effectiveness of each random search strategy was evaluated under the consideration of the aforementioned factors, both intrinsic (motion drift, energy and memory limitations) and extrinsic (physical obstacles and search boundaries). As a first metric for the analysis, we considered the effective length of each travel motion. In the context of sparse targets, Lévy searches are effective when the travel length probability distribution is heavy-tailed^1,34,35^. This was represented as the complementary cumulative distribution function of travel length, which emphasises differences by the Lévy parameter $\alpha$ and other intrinsic and extrinsic factors (Figs. 2 and 3).

**Figure 4:**
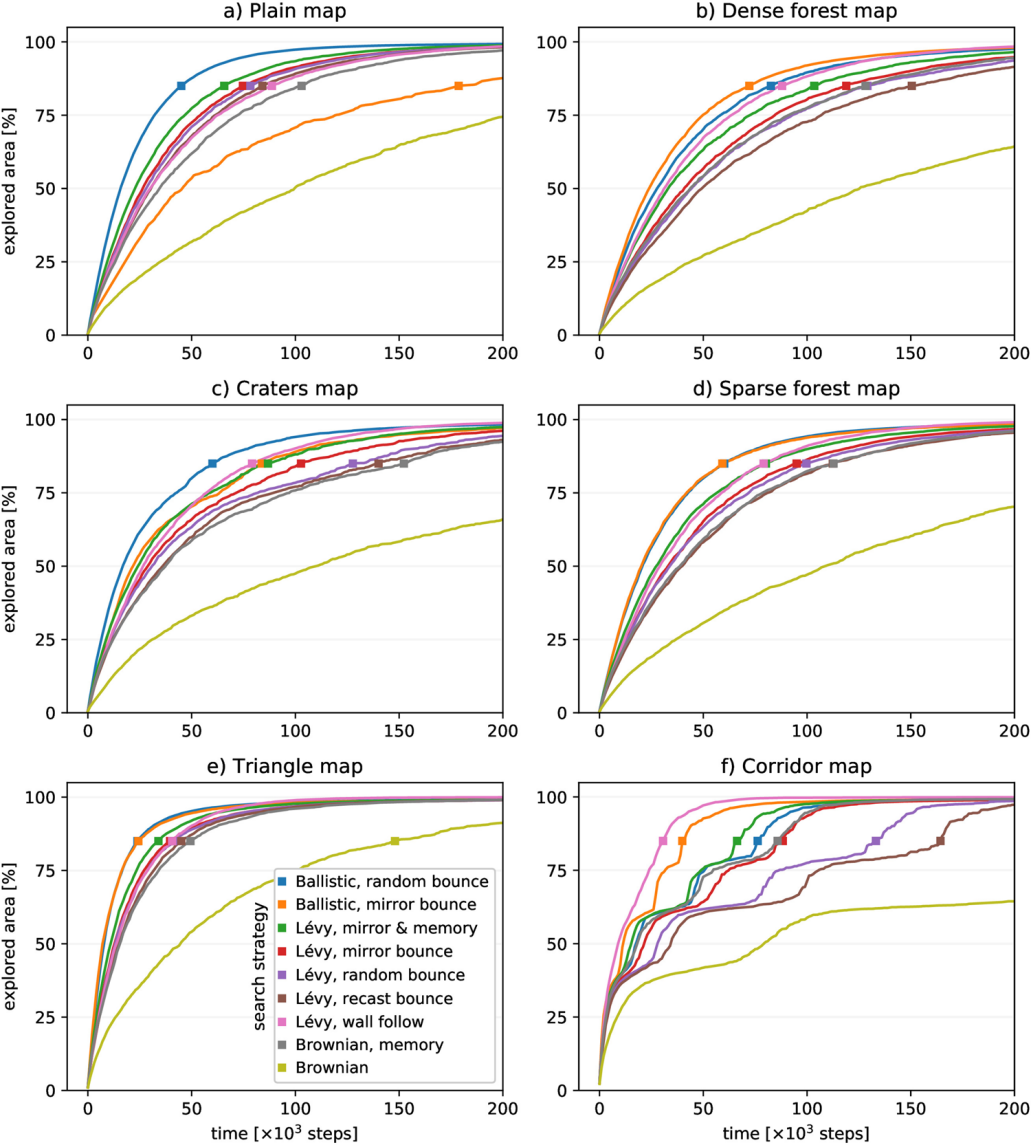
Exploration diffusivity represented as the area explored by each strategy over time. Each trace aggregates 1000 simulations by representing the minimum area covered by at least 900 of the runs at each point in time. A square marker highlights the point where each strategy covered 85% of the total available area (see Table 1 for the area sizes).

The effect of extrinsic factors on the random search can be better analysed when considering the physical reality of the searches and their performance in different environments. As a second metric, we studied the diffusivity or how each strategy develops in time. For this we measured the area explored during the performance of each search on each map. The resulting plots show time in the horizontal axis and the cumulative explored area in the vertical axis. With this data we could directly compare the time performance of the distinct strategies in different domains (Fig. 4).

**Figure 5:**
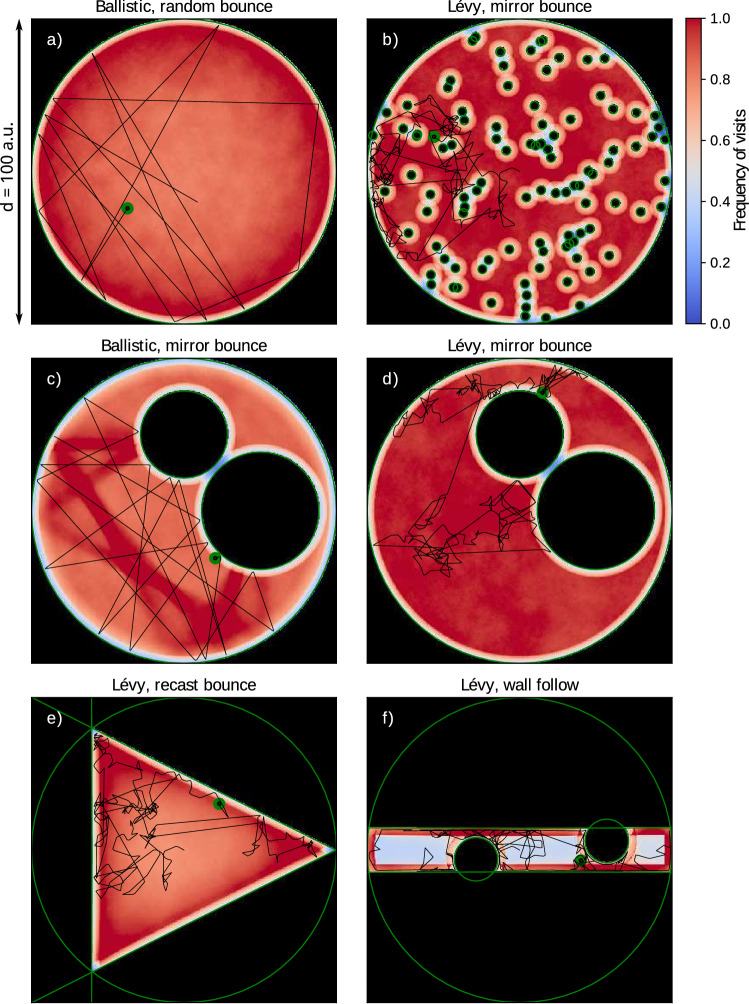
Exploration redundancy represented by heat maps of revisit frequency for a set of search strategies and environments. Panels correspond to: (**a**) Plain map and ballistic random bounce, (**b**) Dense Forest map and Lévy mirror bounce, (**c**) craters map and ballistic mirror bounce, (**d**) Craters map and Lévy mirror bounce, (**e**) Triangle map and Lévy recast bounce, (**f**) Corridor map and Lévy wall follow. Representative trajectories are depicted in black for each map ($10^4$ steps). The supplementary material includes a video animation (Supplementary Video 1) depicting the evolution of this metric for the Craters map with strategies: Ballistic random bounce, Lévy mirror bounce, Brownian with memory, and uninformed Brownian.

As a third metric, we represented the search regions that were most frequently visited by each strategy. This is useful to illustrate the differences in spatial search redundancy. For this goal, we represented the 2D position histogram in the form of heat maps where the colour gradient indicates how frequently each region was visited. This metric is related to the probability distribution on where to find the robot at any moment during the search (Fig. 5).

**Figure 6:**
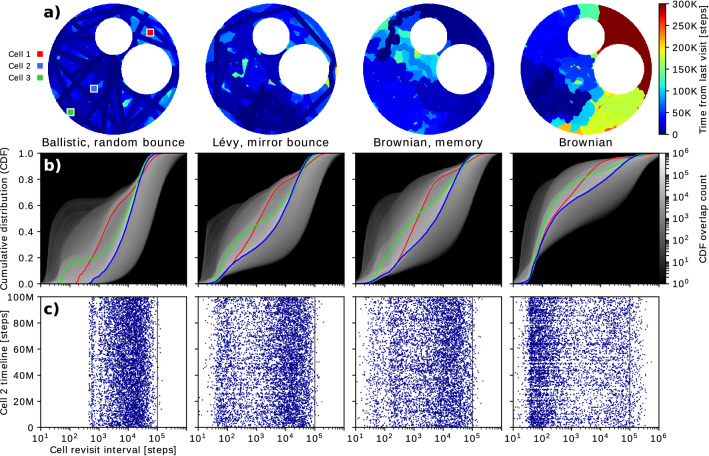
Revisit time analysis. For illustrative purposes we focus our analysis on the Craters map and four search strategies that have different exploration diffusivity levels (see Fig. 4). (**a**) A colour map representation of the time since last visit to each cell at step number 300K. An animated version of this representation (Supplementary Video 2) is also provided. Note the non-uniformity of the Brownian strategy in this representation. Markers 1, 2 and 3 indicate the location of three representative cells which are discussed below. The revisit sequence was also stored for every cell during a 100M step simulation for each strategy. (**b**) Cumulative distribution function (CDF) of the revisit intervals for every cell. There are three overlayed traces corresponding to the CDF for cells 1, 2 and 3. The ballistic strategy shows significantly increased redundancy near the search area limits (cell 3). (**c**) Revisit sequence for cell 2 on a plot where each data point is the revisit interval annotated at the corresponding simulation time step. The heterogeneous revisiting time of the Brownian strategy becomes apparent as it is the only strategy with revisit times $>10^5$ (indicated by a vertical line) which translate into empty horizontal stripes. This effect can be overcome with the implementation of a short-term memory to reduce local repetitions (“Brownian, memory”), which yields similar temporal profile to the Lévy-mirror bounce strategy for this map. The ballistic strategy yields medium revisit times concentrated between $10^3$ and $10^5$.

To analyse the temporal profile of the strategies, we measured the revisit intervals and their evolution for each cell in the environment map. We also performed an analysis of the sequentiality of the revisit events for representative cells and calculated the cumulative distribution functions for the revisit intervals (Fig. 6).

Finally, to favour an intuitive interpretation of these analyses, we also provided two video animations that illustrate the spatial profile (Supplementary Video 1) and temporal profile (Supplementary Video 2) for a set of search strategies on a representative map.

## Results

Intrinsic and extrinsic factors, often disregarded in low-informed exploratory robotics, do distinctly and largely modulate the effectiveness of search strategies under uncertainty. Here we report the results on the influence of such factors over the effectiveness of a random search using the metrics described in the previous section.

### Search diffusivity parameters and factors

The motion pattern of a random search strategy can be modeled as a sequence of displacements or travels in different directions. Superdiffusive strategies such as Lévy search define the length of these travels with a probability distribution that is heavy tailed. Different choices of $\alpha$ (“Robot simulation models” section) define the search diffusivity. In practice, real world factors inherent to robotics often introduce deviations from the theoretical distributions, and thus condition the outcome of a specific choice of the $\alpha$ parameter. Intrinsic factors such as energy limitations or motor plant drift, and extrinsic factors such as obstacles or boundaries in the environment, often result in an overall reduction or modulation of search diffusivity as it is illustrated in Fig. 2.

Blue, green and orange traces in Fig. 2 illustrate the modulation of Lévy parameter $\alpha$. A large value of $\alpha$ ($>3$) in a search strategy driven by model (2) would produce searches with shorter travels, ultimately converging into a Brownian motion implementation. A lower value of $\alpha$ increases the overall diffusivity of the Lévy search, and as a baseline for our study the $\alpha$ parameter was set to 2.5.

Travel paths are also constrained by other intrinsic and extrinsic factors. In the case of environment constraints, travels were truncated by obstacles and boundaries within the search area (purple trace in Fig. 2). In the cases where there is uncertainty in the motor plant, travel truncation arises indirectly from motion drift (brown trace), which distorts the linearity of the paths and affects diffusivity. Finally, in the case of energy limitations it is convenient to truncate longer travels to prevent having a statistics dominated by a single motion. This is illustrated with the “battery truncated” case (red trace in Fig. 2) where travels that are longer than 10% of the total available energy are summarily truncated. Theoretical analysis allows assessing each factor independently of one another. However, in practice, these factors appear in combination.

In Fig. 3 we can observe the combined effect of drift and battery constraints. Starting with the “battery truncated” case (bright-green trace) and considering a small amount of drift (bright-red trace), we can see that differences are moderate: drift effects are being masked by the battery truncation effects. When drift is increased (bright-blue trace), its effect on the diffusivity becomes significant. In fact, drift is now masking the effect of battery constraints.

Beyond its use in search strategy parameterisation, this analysis allows effective decision making over the robotic platform itself, for example to determine whether to dedicate more efforts into improving battery range or whether to improve the locomotive plant first. Thus, the understanding of the effect of these factors can help in the design of a bioinspired random search strategy and, in particular, in the choice of its parameterisation.

### Search area boundaries and obstacles

In search problems under high uncertainty, environmental conditions can be largely undetermined. However, issues such as the maximum exploration range or the presence of obstacles may be estimated or partially known in advance. This knowledge can also include the definition of virtual boundaries to restrict the search. Basic a priori understanding of the effect of such environmental factors can help on the choice of random search strategy.

In this section, we study the effect of the exploration area geometry and the presence of obstacles or inaccessible/forbidden regions. For the sake of simplicity, we focused our analysis on the stereotyped set of environments listed on “Search environments” section. This set is representative for a wide variety of cases of search under uncertainty. In the following subsections, we will analyse the exploration diffusivity and the spatial and temporal profiles of the random search.

#### Exploration diffusivity analysis

Intrinsic and extrinsic modulations result in different evolution of the searches as determined by the area explored over time. The stochastic nature of random search strategies could undermine the representativeness of individual simulation runs on specific scenarios. Thus, to quantify the performance we considered 1000 simulations for each strategy-map combination. Each trace in the panels of Fig. 4 corresponds to the minimum area covered by 90% of the simulations at each point in time. This metric corresponds to the timeline for area coverage, and it allows us to directly compare the search evolution of every strategy under each map. Panels in Fig. 4 also display a square marker representing the point where each strategy covered 85% of the total explorable area. In particular, we compared the maps: Plain, Dense Forest, Craters, Sparse Forest, Triangle and Corridor, while running on them a set of random strategies: Ballistic (with mirror and random bounce), Lévy (with mirror bounce, mirror bounce and memory, random bounce, recast bounce, and wall follow) and Brownian (memory informed and uninformed). The rationale guiding this specific choice of add-ons was to provide a representative set that can illustrate the concepts presented in this paper without clouding the results with too many parameters. More combinations adapted to specific problems can be evaluated using the provided software tools (Supplementary information).

Fig. 4 illustrates how each combination of environment and intrinsic parameters results in a distinct evolution of the search performance. The uninformed Brownian search strategy (yellow trace in the panels) provides a slow search evolution. Ballistic strategies with mirror and random bouncing provide a fast evolution to cover the explored area (orange and blue traces, respectively), granting the best performance with this metric in many maps. However, they can perform poorly in environments with specific geometries, i.e., Ballistic mirror bounce in plots a) and c), and Ballistic random bounce in f). Lévy strategies provide intermediate search evolution speeds. The inclusion of a basic memory improves the exploration time of both Brownian and Lévy strategies in all maps (grey and green traces, respectively). The corridor map has a unique environment condition with three distinct regions and narrow passes in between. Panel f) reflects this, showing “steps” of increasing performance at the times when the robot crossed a passage and explored the different areas of each new region. In this distinct case, the Lévy walk with wall follow naturally provides the fastest exploration (pink trace).

Note that the representation used in Fig. 4 does not provide information about the homogeneity of the exploration for each environment. Thus, it does not allow identifying, for example, those strategies that yield a fast exploration of the environment with a desirable spatial or temporal profile. This is studied in the next sections.

#### Spatial profile analysis

To compare the effect of environment bounds and obstacles over each strategy, we employed a 2D heat map representation that emphasises the regions where searches persist more often. Figure 5 depicts this analysis, highlighting the spatial profiles of a representative set of search strategies and environments.

The analysis in Fig. 5 was implemented with the 2D position grid described in “Robot simulation models” section. Each grid cell stored the total revisit count, i.e., the cell values were updated every time they fell within the robot’s sensory range. Simulations were ran for $10^8$ steps to ensure statistical relevance, and the resulting heat maps were normalised with the maximum and minimum cell visit count. An animated version of this analysis is provided in the Supplementary Video 1 and shows the evolution of the spatial exploration metric for the Ballistic random bounce, Lévy mirror bounce, Brownian memory, and Brownian strategies.

The heat maps in Fig. 5 represent the spatial search profile, which is related to the probability of finding the robot in a particular location (time considerations are studied in the next section). Panel a) corresponds to the Plain map and a ballistic random bounce search, where the exploration becomes emphasised near the concave boundary. The increased probability is due to the convexity of the walls, which concentrates the 180$^\circ$ probability, i.e., there is a slightly higher chance for the robot to go against the sides of the collision point (the same effect can also be observed in the Craters map, see Supplementary Video 1). Panel b) depicts the Dense Forest map and a Lévy mirror-bounce search. Despite the complicated map, the strategy achieves a highly homogeneous exploration arising from the Lévy diffusivity in combination with the effectiveness of mirror bounce. Panels c) and d) compare the performance of two strategies in the Craters map: c) depicts the ballistic mirror-bounce search, where heterogeneity arises when this particular strategy finds symmetries in the environment, and d) depicts the Lévy mirror bounce strategy, where the exploration is again highly uniform (see also Supplementary Video 1). Panels e) and f) incorporate different geometries: e) is the Triangle map and a Lévy strategy with recast bounce, with a dense exploration near the corners due to the truncation of the Lévy step upon each collision, and f) is the Corridor map and a Lévy wall-follow search that favours the passage through narrow areas while yielding less exploration in regions far from the walls.

Boundaries can have different effects depending on the implemented search strategy and avoidance routines. There is often less redundancy right at the boundaries (the only exception is the “wall follow” obstacle handler) and this is related to sensor diameter range. Strategies a) and e) display a tendency towards boundaries that could be useful in some scenarios. Ballistic strategies can result in fast but otherwise non-homogeneous exploration, highly dependent on the specificities of the environment, as shown in panels a) and c). Note that Lévy mirror bounce strategies that yielded intermediate exploration diffusivity (“Exploration diffusivity analysis” section) can result in a largely homogeneous spatial exploration, cf. panels b) and d) and Fig. 4.

Our work with these examples shows that the presence of obstacles and search area restrictions, in combination with the chosen avoidance strategies, can have a significant effect on the spatial uniformity of the random searches. According to the results, specific environmental conditions may require a simple modulation of the search strategy to satisfy the particular spatial uniformity requirements.

#### Temporal profile analysis

We also performed an analysis of the revisiting time profile during the evolution of the distinct strategies. It is important to note that the ability of a given strategy to provide a particular revisiting profile is not fully represented in Figs. 4 and 5. Search problems under uncertainty which consider latency or movement of the targets may want to take into account or favour homogeneous temporal revisiting, or to avoid revisiting in specific intervals, etc. (see^36^). This analysis is shown in Fig. 6 for the Craters map and a selection of four representative strategies: Ballistic random bounce, Lévy mirror bounce, Brownian memory, and Brownian. A second video animation (Supplementary Video 2) allows us to better illustrate the temporal profile analysis, as it displays the revisit intervals and their evolution in time.

Panel a) in Fig. 6 shows a snapshot of the temporal distribution of each exploration strategy at step 300K. Dark blue colours indicate a recent visit, whereas brighter/red colours correspond to longer delays. Different patterns indicate variations in the revisiting profile of each search strategy. Searches that display uniform blue colours produce relatively frequent revisits with different spatial patterns. The presence in this representation of distinct colour patches is indicative of larger temporal heterogeneity. It is the case of Brownian search: though it can yield uniform spatial coverage as illustrated in the first video animation (Supplementary Video 1), it is notably heterogeneous in terms of the time since last visit.

Panel b) in Fig. 6 shows the cumulative distribution function (CDF) of cell revisit intervals, for the same strategies depicted in panel a) and Supplementary Video 2. The revisit times were stored at each cell for every visit during the 100M step simulation. The CDF was then computed for every grid cell, and each CDF trace was aggregated to create the shaded plots in the panel (brighter traces indicate the most frequent probability distributions). Three representative cells were selected and their respective CDFs highlighted in colour. Cell 1 (red) is located at the region between the craters and the nearest search boundary, cell 2 (blue) is at an area away from obstacles and bounds, and cell 3 (green) is located near the search boundary away from the craters. Note the different temporal profile for the different strategies and for the three representative cells. In particular, the step-like evolution of cell 3 -in contrast with cell 2 for the ballistic search- highlights the shorter revisit times near the boundaries for this particular strategy.

Panel c) in Fig. 6 displays the sequence of revisit times for cell 2, each data point corresponding to a revisit interval annotated at the occurring simulation step. This panel shows that the ballistic strategy is most affected by the search area size and shape, with an unpopulated revisit range near $10^2$ that is defined by the cell-to-boundary distance. The incorporation of a local memory to the Brownian strategy results in two distinct effects: there is less density near $10^2$ and there are less revisits above $10^5$. This suggests that the temporal profile can be intentionally modulated with a local memory and its parameterised size to achieve a specific revisiting time profile.

The knowledge of target latencies, together with the effect of search parameters and intrinsic and extrinsic factors on the revisit temporal profile, can be used to optimise a random search strategy. Our work presents a novel method for the spatial and temporal analysis of these strategies. Combined with diffusivity requirements and the distribution of the search targets, this methodology can be employed to satisfy the spatial and temporal characteristics of a search under a specific problem and uncertainty level.

## Conclusions

The results described in previous sections allow extracting several recommendations when designing a random search strategy for low-informed problems to modulate the strategy with basic a priori knowledge. In this section we summarise our main conclusions.

### Identification of factors and associated parameters to optimize random search under uncertainty

Random search under high uncertainty is often implemented in different contexts as simple Brownian and Lévy strategies^37^. The identification of factors and associated parameters that contribute to minimize redundancy of the search and adapt to the specificities of the considered problem leads to optimization. In particular, the study of the influence of the parameters in the diffusivity and temporal properties of the search helps in selecting the random strategy, its modulation, and the associated implementation on a given platform. A strategy that theoretically endows large diffusivity can be largely truncated by implementation constraints such as drift or battery life (cf. Fig. 3). Taking into account such truncation with the techniques presented allows for a better parameterisation of the chosen strategy.

The quantification of both individual and joint contributions of long-term motion drift, battery capacity, random strategy parameters and their tolerable range, favor the decision on which choices yield better results for the resources available. For example, this quantification can indicate that it is more efficient for the random search goal to first improve the motor plant drift than to extend the battery life (as bright-blue and bright-red traces show in Fig. 3). This combined parameterisation is crucial to achieve a higher search efficiency, even with very little information available.

### Considerations for bounded environments

The design of robust obstacle-handling reactions is of special relevance in uninformed search problems. As shown in Fig. 4, a wrong choice in this aspect can impair the performance of specific strategies to the point of reducing their inherently high diffusivity. When interacting with the specific features in the environment, the bounce strategies can modulate the overall search uniformity (cf. Fig. 5). While there is no universal low-informed strategy that is optimal for all cases, basic known characteristics of the search area hint the modulation for an effective choice of the boundary and obstacle handling approaches. This includes the parameters of intrinsic factors that define adaptation of the motor plant to the environment. It is important to emphasize that modulation with the discussed approach also allows us to define a more selective exploration that better matches target distribution, i.e., defining a preference for enclosed regions, avoiding the areas near map borders, providing a more effective exploration of narrow passages, etc.

Regarding the spatial distribution of the different searches, the Ballistic strategy and the Ballistic random bounce strategy display the largest exploration diffusivity for all maps in Fig. 4 except for the corridor map. For this later type of restricted environments, the Lévy wall follow strategy provides the best performance.

### The importance of revisit time distributions

The evaluation of the spatial and temporal homogeneity of random searches is useful from many different perspectives. In most situations, a homogeneous average spatial revisiting of the search area is a desirable property. However, spatial redundancy analysis does not provide a complete characterisation on its own. For instance, search problems under uncertainty which consider latency or movement of the targets may want to favor an homogeneous *temporal* revisiting, or to avoid revisiting in specific intervals, etc. We provided a comprehensive study of the revisit intervals, their probability distributions and their variability over time for illustrative cases. The analyses shown in Fig. 6 and video animations (Supplementary Video 1 & 2) indicate that this is possible even under low-informed random searches.

Note that in Fig. 6 and specifically row c), for the Ballistic random bounce strategy, a target located at Cell 2 on the Craters map would be favorably revisited in the $\approx 10^4$ interval range. With the Lévy mirror bounce strategy the distribution turns bimodal instead, and revisits are in the $\approx 10^2$ and $\approx 10^4$ interval ranges. With the Brownian memory strategy there is a similar situation at first glance, but we can now observe a distinct gap at the $\approx 10^2$ interval - such revisit interval is effectively being avoided. Finally, with the regular Brownian strategy revisits in the interval $\approx 10^2$ are now strongly favored, and additionally this strategy involves larger revisit intervals ($>10^5$). Should we need to cover the full range of revisit intervals during the search, one possibility would be to combine the speed of a Ballistic random bounce strategy with the baseline latencies of a plain Brownian search, for instance by alternating between each behaviour over time. The knowledge of revisit intervals of a specific random strategy, and in particular the associated range and distribution as shown in this study, is also highly relevant towards an accurate estimation of latency distribution for targets in the context of robotic search problems.

### Modulation adding up basic revisit memory

Incorporating a simple short-term spatial memory reduces local redundancy, and can add up to to basic intrinsic and extrinsic modulation strategies of the random search (Figs. 1, 4, 6), see also^38^ for its effect on patchy environments. The Lévy mirror & memory strategy has more exploration diffusivity than Lévy strategies without memory when environments do not have a large number of obstacles. However, in environments with many obstacles, the Lévy wall follow strategy is closer to the memory-based Lévy regarding exploration diffusivity. Memory is of course limited in those cases where external reference systems are not available or when drift is present. Adjusting the size of the memory allows us to adjust/optimize the revisit frequency (specifically to prevent revisits in a desired time interval) when latency of the targets is known. Memory has a stronger effect in strategies that produce frequent local redundancy by making them more diffusive (cf. Fig. 1). Memory also shapes revisiting time profiles. The techniques presented in our study allow to analyze and selectively adjust the spatial and temporal characteristics of a search, even under uncertainty.

## Discussion

There are many examples of animal search behaviour that can be described by random searches^39–41^. Animals however, modulate at any time their search strategies with partial information provided by their sensory systems, which typically have different ranges and sensibilities. Modulation also comes from the internal representation of the search within their brain and the associated embodiment^42^ balancing energy resources and search goals.

Bioinspiration in robotics can come from specific aspects of biological motor plants and locomotion control^43–45^, but also from how information availability and uncertainty is handled by the nervous system. In autonomous robotic search under uncertainty, modulation provided by the low information available results in optimised random searches. We have illustrated this by analysing distinct random searches and their modulation as a function of basic specific knowledge of intrinsic and extrinsic factors that affect the robot and its motion.

Modulation of intrinsic and extrinsic factors in random search exploration under high uncertainty is relevant in a wide variety of environments (e.g., in cave, deep ocean and planetary exploration), and also in situations where bioinspired strategies could be a robust option (e.g., upon sensor interference or signal loss). Aspects such as locomotion drift, power estimates, basic boundary/environment information, which may be known a priori, can be readily employed to modulate a random search under uncertainty for optimisation purposes. This modulation can be considered as a simple implementation of embodiment^46,47^, as a way to incorporate into the strategies the intrinsic limitations from the mechanical plant or from the sensors, or available information from the environment. High levels of uncertainty demand that any knowledge regarding the robot itself, the environment and/or their interaction, no matter how basic, is used in the search strategy. In many cases this information can be added with little computational cost^21,48^.

In this paper we have shown that modulation by intrinsic and extrinsic factors affect both the spatial distribution and diffusivity of the exploration, as well as the profile of the revisiting time. Both aspects are highly relevant and worth considering when designing low-informed robotic strategies in a wide variety of target conditions: static or moving targets, targets displaying latency when appearing or disappearing, targets with latency affected by the revisits, etc. Our results using representative examples indicate that basic knowledge from the modulation can be incorporated into the strategies to make them more effective and adapt them to the specific context of a random search problem. We expect these results to be valid for a wide variety of problems related to the identification of factors and associated parameters to optimize random search under high uncertainty, particularly on the considerations for bounded environments, the importance of revisit time for specific applications, and the modulation adding up basic revisit memory. The proposed approach could also be extended to tackle other parameters such as variations in search area size, the incorporation of an obstacle memory, inter-robot cooperation, assessment of sensory range latency, etc. Our simulation tool is provided to facilitate the analysis under other variations of the problem.

Effective hybridisation of strategies^38,49^ can also be viewed as a bioinspired approach to autonomous search under uncertainty, with the modulation coming from sensory range information switches.

The results reported in this paper suggest multiple means to such kind of implementations, which can take advantage of the software platform provided with this paper. In the context of multi-robot searches, swarm search strategies under uncertainty can also largely benefit from the combination of low-knowledge modulation approaches discussed above.

## Supplementary Information

Supplementary Information.

Supplementary Video 1.

Supplementary Video 2.
