# Supplementary material for: Intrinsic and environmental factors modulating autonomous robotic search under high uncertainty
Source: Sci Rep. 2021 Dec 31;11:24509. doi: 10.1038/s41598-021-03826-3 (PMC8720098; doi:10.1038/s41598-021-03826-3)
Supplement: Supplementary file 1 — Supplementary Information. [file 41598_2021_3826_MOESM1_ESM.pdf]

Intrinsic and environmental factors modulating autonomous robotic search under high uncertainty

Carlos Garcia-Saura[1,*], Eduardo Serrano[1], Francisco B. Rodriguez[1], and Pablo Varona[1,*]

[1]Grupo de Neurocomputación Biológica, Dpto. de Ingeniería Informática, Escuela Politécnica Superior, Universidad Autónoma de Madrid, 28049 Madrid, Spain

[*]carlos.garciasaura@uam.es, pablo.varona@uam.es

Supplementary material

Supplementary Video 1: *Animation of the spatial revisit profile.* This video illustrates the evolution of exploration redundancy for four representative search strategies on the Craters map: Ballistic random bounce, Lévy mirror bounce, Brownian with memory, and uninformed Brownian. Each strategy was simulated for 100M steps and the visit count was monitored for every location on the search area. The heat map representation and colour are the same used in Fig. 5 and indicate the revisit density throughout the search area. Video pauses were incorporated at steps 1K, 10K, 100K and 1M to facilitate comparing between strategies. The video highlights the evolution of the spatial profile among strategies, and the final overall spatial distribution of the explorations.

Supplementary Video 2: *Animation of the temporal revisit profile.* This video depicts the evolution of the time since last visit for the same four strategies and maps that were illustrated in the spatial revisit profile animation (Supplementary Video 1). The strategies were simulated for 1M steps while annotating the last visit for every point on the search area, and the video shows how the revisiting evolves throughout the simulation. The heat map representation and colour scale are the same used in Fig. 6 and indicate the time since last visit. The video highlights differences in diffusivity and temporality among strategies, with an emphasis in the temporal properties of the exploration.

Supplementary information 3: *Software repository.* Our simulation platform is provided for an easy reproduction of the results and for its use when exploring other modulating intrinsic and extrinsic factors to optimize search strategies under uncertainty in specific environments:

`https://github.com/GNB-UAM/RoboSearcher`
